# Oral Blue Nevus of the Lingual Gingiva: A Case Report

**DOI:** 10.7759/cureus.77405

**Published:** 2025-01-13

**Authors:** Hamad Albagieh, Khalid W Alrashed, Mohammed Y AlGammlas, Hassan Khormi, Fares M Hodan, Mohammed F Alem, Faisal Alhelal, Abdullah M Albaiz

**Affiliations:** 1 College of Dentistry, King Saud University, Riyadh, SAU

**Keywords:** blue nevus, oral histopathology, oral medicine, oral melanocytic nevi, pigmented lesions

## Abstract

Oral melanocytic nevi (OMN) are rare benign tumors originating from melanocytes with an unclear pathogenesis. The current theory suggests that OMN originate from dormant dendritic melanocytes that become enclosed in the dermis during the embryonic migration of melanoblasts - the precursors of melanocytes - from the neural crest to the epidermis. OMN can be congenital or acquired, with acquired nevi being more common. These nevi are classified into several types based on histological features, including junctional, compound, intradermal, combined, and blue nevi. Among these, intramucosal nevi are the most frequent, followed by blue nevi. Oral blue nevus, although rare, is typically asymptomatic and commonly found on the hard palate. This report presents a case of a blue nevus located on the lingual attached gingiva of a 37-year-old male patient. Despite the benign nature of blue nevi, their clinical appearance often mimics other pigmented lesions, including malignant melanoma. This case highlights the importance of distinguishing blue nevi from other oral melanocytic lesions through histopathological examination to ensure proper diagnosis and management. The report also emphasizes the role of routine oral examinations in the early detection of such lesions, especially given the potential for misdiagnosis in clinical practice.

## Introduction

Oral melanocytic nevi (OMN) are uncommon, benign tumors originating from melanocytes [1]. Their pathogenesis and etiology remain poorly understood [1]. The current theory suggests that OMN originate from dormant dendritic melanocytes that become enclosed in the dermis during the embryonic migration of melanoblasts - the precursors of melanocytes - from the neural crest to the epidermis [1-3]. OMN may be either congenital or acquired, with the latter being more prevalent [1]. Histologically, they are classified into junctional, compound, intradermal (intramucosal), combined, and blue nevi based on the location and formation of nevus cells [1,2]. Among these, the intramucosal nevus is the most common, and then comes the blue nevus [4].

Oral blue nevus is rare, with a prevalence of approximately 0.1% in the general population [4]. It is classified into two main types: the common blue nevus and the cellular blue nevus (CBN) [5]. It typically manifests between the third and fifth decades of life and is more commonly observed in women than in men [5]. The most common site is the hard palate, after which is the labial mucosa and then the vermillion border [1,4]. These lesions are usually asymptomatic and are often discovered incidentally during daily dental examinations [1,4].

Clinically, the usual blue nevus appears as an isolated, smooth-surfaced, well-defined papule or nodule, which can be either flat or elevated and typically measures less than 10 mm in diameter, even though bigger lesions have been documented [1,4-6]. CBNs usually present as pigmented nodules ranging from a few millimeters to several centimeters in size [5]. The color ranges from brown to blue, based on the amount of pigmentation, depth, and site [1,4,5].

Histologically, the hallmark of the blue nevus is a pigmented, spindle-shaped dendritic melanocyte with slender, branching dendritic processes, lacking atypia or mitotic activity [1,4,5,7]. The nuclei are small, elongated, and hyperchromatic [1,4,5,7]. Blue nevus cells are commonly positive for S100, MelanA (MART-1), and HMB-45 [1,4,5,7]. Although it is rare, it has been reported that there is a chance of malignant transformation of CBN into oral malignant melanoma (OMM) [5].

The aim of this report is to present a rare case of an oral blue nevus located in the lingual attached gingiva, highlighting its clinical presentation, the diagnostic challenges it poses, and the critical importance of distinguishing it from other pigmented oral lesions to ensure accurate diagnosis and effective management.

## Case presentation

A 37-year-old male was referred to the Oral Medicine Clinic at the Dental University Hospital, King Saud University, with a pigmented nodular lesion located on the lingual attached gingiva of the lower left side, adjacent to teeth #34 and #35. The patient reported that the lesion had been present for five years without any changes in size. He did not experience pain or any secondary symptoms associated with the growth.

The patient's medical history was significant for depression and chronic sinusitis. He was on citalopram, a selective serotonin reuptake inhibitor (SSRI), for depression and pseudoephedrine, an antihistamine, for sinusitis. He is a former smoker with a 15-year history of smoking one pack per day, who has since transitioned to smoking sheesha (Hookah) twice daily. There was no contributory family history.

**Figure 1:**
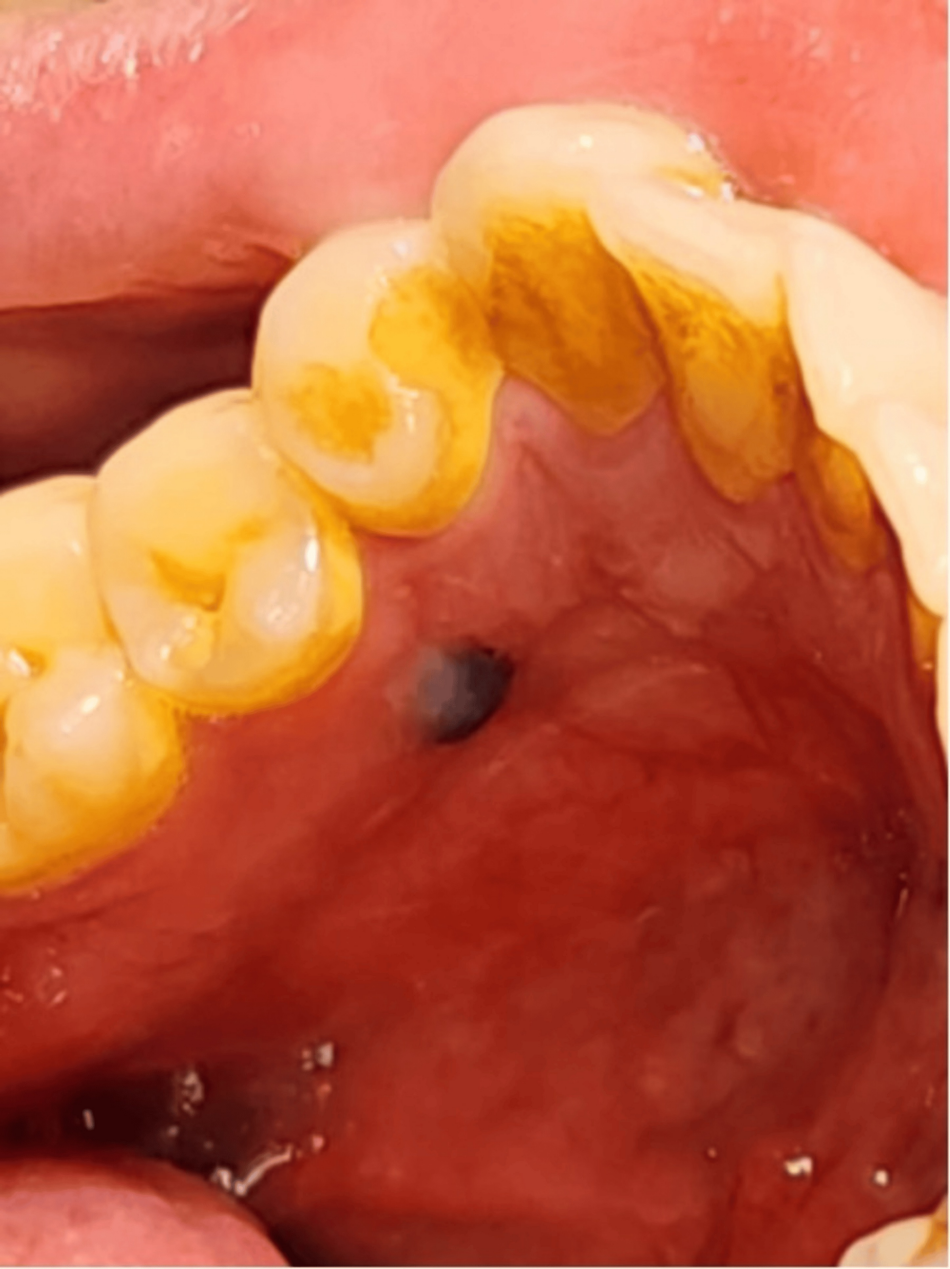
A 0.3 × 0.3 cm round, raised, black lesion was observed on the attached gingiva, lingual to teeth #34 and #35.

**Figure 2:**
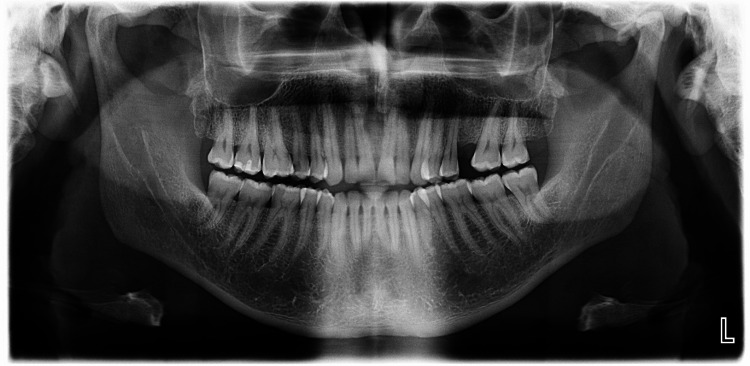
Panoramic radiograph.

Upon intraoral examination, a 0.3 × 0.3 cm round, raised, homogeneous black lesion was noted on the attached gingiva lingual to teeth #34 and #35 (Figure 1). The lesion was asymptomatic, with no associated tooth mobility or deep periodontal pockets upon probing. Radiographic examination, including a panoramic radiograph, showed no abnormalities, and no osteolytic lesions were observed (Figure 2).

**Figure 3:**
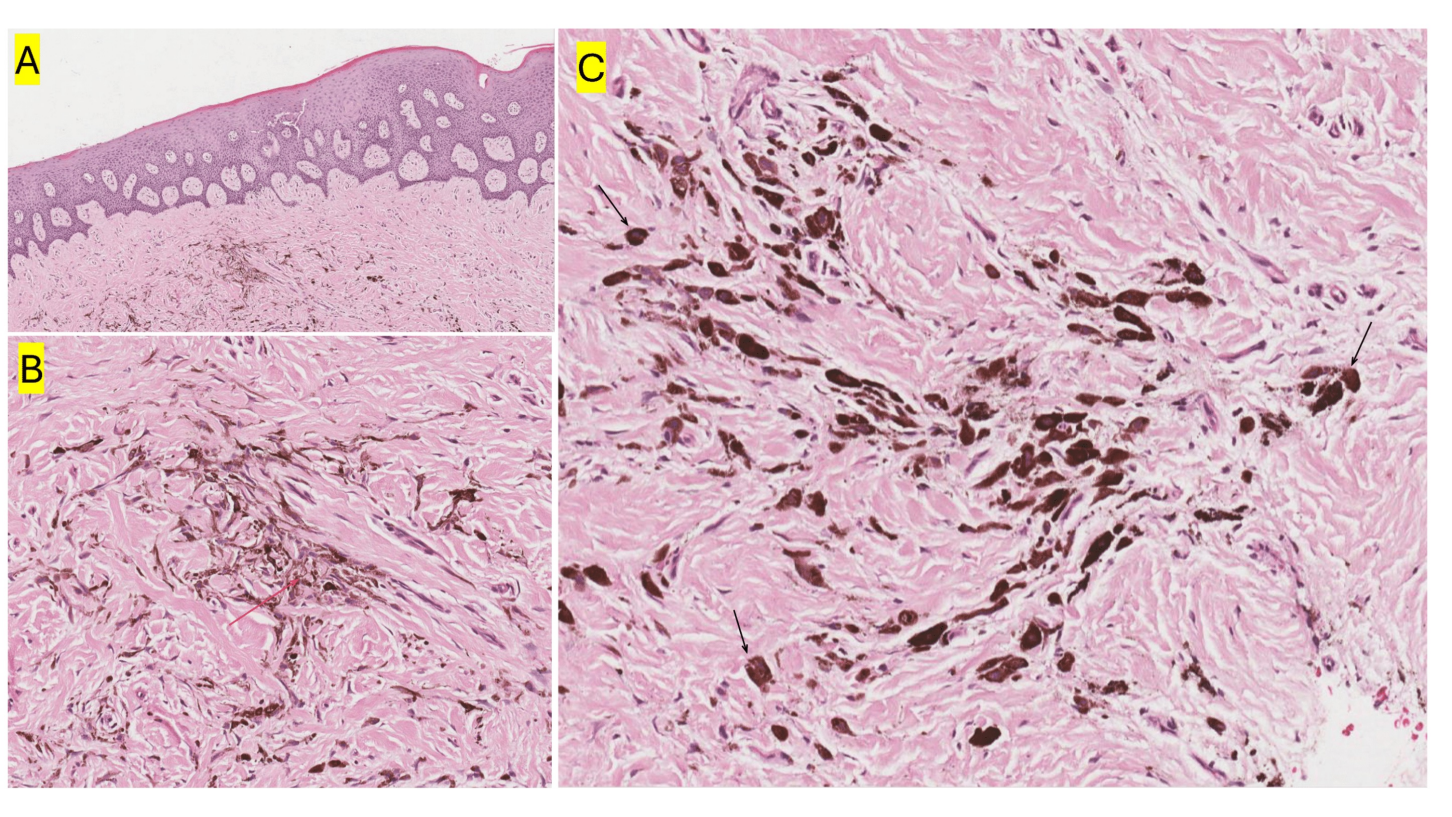
(A) Low power view illustrating a fragment of oral mucosa surfaced by keratinized stratified squamous epithelium, with pigmented cells within densely collagenized fibrous connective tissue aligned parallel to the surface epithelium. (B, C) Higher power views illustrate pigmented spindle-shaped melanocytes, some of which are dendritic (red arrow), along with epithelioid melanocytes (black arrow).

Based on the clinical presentation, the differential diagnosis includes oral melanotic macules, OMN, and OMM. To establish a definitive diagnosis and rule out OMM, an incisional biopsy was performed under local anesthesia. After obtaining written and verbal consent, a safety pause was conducted to ensure that all necessary precautions were in place. Hemostasis was achieved successfully using gauze and pressure, with an estimated blood loss of less than 1 cc. The patient was provided with written and verbal postoperative instructions and discharged in stable condition. The biopsy specimen was placed in a labeled container, immersed in 10% formalin, and sent to the oral pathology laboratory for histopathological examination. The histopathological examination revealed a fragment of oral mucosa surfaced by keratinized stratified squamous epithelium with focal basal cell melanosis. The main feature of the specimen was the presence of pigmented spindle-shaped melanocytes, some of which were dendritic, along with epithelioid melanocytes, both aligned parallel to the surface epithelium. These melanocytes were embedded within densely collagenized fibrous connective tissue (Figures 3A-3C). This histopathological finding is consistent with the diagnosis of a blue nevus.

## Discussion

The diagnosis of pigmented lesions in the oral cavity can be complex, as these lesions are often asymptomatic and frequently discovered during routine oral examinations [7]. While the ABCDE (asymmetry, border, color, diameter, evolution) system may serve as a useful tool for assessing the potential malignancy of oral lesions, clinical evaluation alone is inadequate for a definitive diagnosis [7]. As a result, most pigmented lesions are ultimately excised for histopathological examination [7-9].

The term "blue nevus" refers to dark blue lesions typically found on the skin and was first brought into the literature by Tièche in 1906 [10]. The blue-black color of these lesions is primarily attributed to the Tyndall effect, which occurs when melanocytes are located deep within the dermis or the lamina propria of the mucosa [1,4,5,11]. The various shades of blue observed in blue nevi are influenced by several factors [10,11]. The presence of melanocytes in the overlying epithelium or epidermis can alter the color, as can the depth at which these melanocytes are located within the tissue [1,4,10,11]. Additionally, the number of melanocytes containing melanin plays a crucial role in determining the intensity of the blue hue [1,10,11]. Essentially, the greater the number of melanin-containing melanocytes and the deeper their location, the more intense the blue color will appear [1,10,11].

Blue nevus often harbors activating mutations in GNAQ and less frequently in GNA11. Blue nevus is a benign tumor that does not usually recur after complete excision. Recurrences occasionally occur after incomplete excision and usually resemble the original tumor [5].

These lesions are typically asymptomatic and occur twice as frequently in women compared to men. However, in our case, the lesion was identified in a male patient, which is less common [1,5]. Oral blue nevi generally manifests in individuals between the third and fifth decades of life, with an average age of 35 years [1,4,5]. The literature review indicated that oral blue nevus predominantly occurs on the palate [1,4,5]. The exact reason for this site predilection remains unclear, but one possible explanation is the involvement of neural crest cells in the development of the palate [12]. It can be hypothesized that remnants of these neural crest cells, which remain after palate formation, may give rise to both nevi and malignant melanomas [4].

While oral nevi are rarely present at birth or linked to genetic conditions, they are usually acquired later in life due to a mix of genetic and environmental factors. Although common nevi on the skin often show specific gene mutations (BRAF or NRAS), it is still unknown if these same mutations play a role in how nevi develop in the mouth [13,14].

A biopsy is essential for accurately diagnosing an oral melanocytic nevus, as the differential diagnosis for focally pigmented lesions includes malignant melanoma. While malignant transformation has been reported in cutaneous blue nevi, this phenomenon has not been consistently observed in oral blue nevi. Despite this, conservative surgical excision remains the preferred treatment for these oral lesions [15].

In their review of multiple cases of oral nevi, Buchner and Hansen reported the distribution of different clinical variants: the intramucosal type was the most common, accounting for 55%, followed by the common blue nevus at 32%, compound nevi at 6%, junctional nevi at 5%, and combined nevi at 2% [9]. They also examined the incidence of nevi at various intraoral sites, which is summarized in (Table 1).

**Table 1:** Distribution of incidence percentage of oral nevus

Site of nevus	Percentage of incidence
Hard palate	42%
Buccal mucosa	17%
Retromolar pad	11%
Gingiva	8%
Vermillion border	8%
Soft palate	8%
Labial mucosa	3%
Tongue	3%

## Conclusions

This case highlights the importance of recognizing and accurately diagnosing blue nevi in the oral cavity, as they can resemble more serious conditions like melanoma. A biopsy and histopathological analysis are crucial for diagnosis. The case is noteworthy for its rare location on the attached gingiva and emphasizes the need for thorough oral examinations, as such nevi are often asymptomatic. It serves as a reminder for clinicians to remain vigilant with pigmented oral lesions and to perform biopsies when diagnosis is uncertain or serious conditions are suspected.
